# Peripheral Neuroinflammation and Pain: How Acute Pain Becomes Chronic

**DOI:** 10.2174/1570159X21666230808111908

**Published:** 2023-08-09

**Authors:** Mark A. Schumacher

**Affiliations:** 1 Department of Anesthesia and Perioperative Care and the UCSF Pain and Addiction Research Center, University of California, San Francisco, California, 94143 USA

**Keywords:** Chronic pain, inflammation, NGF, sensory neuron, transcription factor, TRPA1, TRPV1

## Abstract

The number of individuals suffering from severe chronic pain and its social and financial impact is staggering. Without significant advances in our understanding of how acute pain becomes chronic, effective treatments will remain out of reach. This mini review will briefly summarize how critical signaling pathways initiated during the early phases of peripheral nervous system inflammation/neuroinflammation establish long-term modifications of sensory neuronal function. Together with the recruitment of non-neuronal cellular elements, nociceptive transduction is transformed into a pathophysiologic state sustaining chronic peripheral sensitization and pain. Inflammatory mediators, such as nerve growth factor (NGF), can lower activation thresholds of sensory neurons through post-translational modification of the pain-transducing ion channels transient-receptor potential TRPV1 and TRPA1. Performing a dual role, NGF also drives increased expression of TRPV1 in sensory neurons through the recruitment of transcription factor Sp4. More broadly, Sp4 appears to modulate a nociceptive transcriptome including TRPA1 and other genes encoding components of pain transduction. Together, these findings suggest a model where acute pain evoked by peripheral injury-induced inflammation becomes persistent through repeated cycles of TRP channel modification, Sp4-dependent overexpression of TRP channels and ongoing production of inflammatory mediators.

## 1 INTRODUCTION

Estimates of the number of individuals suffering from chronic pain in the United States and its physical, emotional, and financial toll are staggering [1]. Despite tremendous clinical advances observed in fields such as trauma care, orthopedics, and oncology, the consequence of survivorship often involves chronic pain and disability. In the case of cancer care, operative procedures and chemotherapeutics may extend life but at the cost of developing a chronic painful condition [2]. This paradox results from the lack of safe and effective therapeutics for the treatment of chronic pain. One of the greatest challenges in developing successful treatments for chronic pain is to refocus research efforts to understand how pain persists well after visible signs of tissue injury have abated.

## 2 MODELS OF ACUTE TO CHRONIC PAIN

Pain arising from peripheral tissue injury and nerve injury is driven by activity in primary afferent nociceptors -those specialized sensory neurons dedicated to the detection of impending or actual tissue injury [3-5]. Depending on the inciting event (inflammation, neurotoxicity), peripheral signaling pathways under inflammatory and neuroinflammatory conditions conspire to amplify and produce the persistence of pain [6-8]. As proposed in Fig. (**1**), there are overlapping components that underly the transition from acute to chronic pain. Inflammation-induced hypersensitivity entails modification of nociceptor ion channel function that results in lowering activation thresholds in the presence of the ongoing production of endogenous sensitizing molecules [5, 9, 10]. Additionally, post-translational modification of receptor channels and parallel transcription-dependent changes in nociceptor gene expression, together, support the transition from acute to chronic pain. For example, studies link inflammation-mediated nociceptor sensitization, post-translational modification and increased expression of nociceptor transient-receptor potential TRPV1 and TRPA1 to profound changes in nociceptor signaling and the persistence of painful hypersensitivity [11-25].

The nervous system may, in fact, be poorly designed to limit or suspend its activity once a noxious stimulus has been removed or the disease process resolved. From the nervous system’s point of view, “routine” recovery from trauma or successful treatment for cancer with chemotherapy surgery may represent unexpected survival. Treating chronic pain as if it is simply another day of acute pain ignores evolving pathophysiologic changes in the architecture of the pain pathway. This approach has been observed on a massive scale with the widespread and often misguided use of opioids for chronic noncancer pain (such as back pain), propelling many countries into an epidemic of opioid misuse, overdose, and death [26]. Alternately, developing therapeutics that target plasticity changes in the peripheral nervous system, the site of pain transduction and transmission, may hold promise to reverse the neuroplasticity changes that drive persistent pain states. Such an approach may succeed without the recruitment of central nervous system reward centers driving addictive behaviors.

Mechanisms describing the transition from acute to chronic pain are largely based on the study of preclinical rodent models of painful hypersensitivity and include, Supraspinal Modulation: Alterations in signal processing above the spinal cord, especially changes in plasticity dedicated to descending inhibition of second-order dorsal horn neurons [8]; Central Sensitization: Abnormalities of nociceptive signaling and plasticity in the spinal cord dorsal horn that enhance facilitation of nociceptive neurotransmission and are dependent on N-methyl-D aspartate (NMDA) - mediated signaling [27]; Peripheral Sensitization: Development of persistent dysfunction of the peripheral nervous system including the dorsal root ganglion often typified by lowering nociceptor activation thresholds or resulting in spontaneous nociceptor activation [28]. Neuroinflammation, the recruitment, and activation of the peripheral nervous system’s innate immune system in response to injury, is proposed to participate across these three nervous system components. This review will focus on the role of tissue inflammation and neuroinflammation in the peripheral nervous system to transition from acute to chronic pain hypersensitivity.

Pain arising from peripheral tissue injury and nerve injury is driven by activity in primary afferent nociceptors - those specialized sensory neurons dedicated to the detection of impending or actual tissue injury [3-5]. Depending on the inciting event (inflammation, neurotoxicity), peripheral signaling pathways under inflammatory and neuroinflammatory conditions conspire to amplify and produce the persistence of pain [6-8]. As proposed in Fig. (**1**), there are overlapping components that underly the transition from acute to chronic pain. Inflammation-induced hypersensitivity entails modification of nociceptor ion channel function that results in lowering activation thresholds in the presence of the ongoing production of endogenous sensitizing molecules [5, 9, 10]. Additionally, post-translational modification of receptor channels and parallel transcription-dependent changes in nociceptor gene expression together support the transition from acute to chronic pain. For example, studies link inflammation-mediated nociceptor sensitization, post-translational modification, and an increase in expression of nociceptor transient-receptor potential TRPV1 and TRPA1 to profound changes in nociceptor signaling and the persistence of painful hypersensitivity [11-25].

## 3 NERVE GROWTH FACTOR AND NEUROINFLAMMATION

Nociceptor modulation is complex, and multiple pathways exist to both detect noxious stimuli and modulate transducing element sensitivity. Under conditions of tissue and nerve injury, the complexity is increased, driven by overlapping biochemical processes - often common to both tissue and neuroinflammation. One signaling molecule long associated with the persistence of neuropathic pain and the modulation of the peripheral nociceptive system is the neurotrophic factor, nerve growth factor (NGF). Since its identification by Levi-Montalcini and Calissano, NGF has been distinguished from other neurotrophin family members (brain-derived neurotrophic factor NT-3 and NT-4/5) as being essential for normal nociceptor development and function [29-31].

NGF is essential for the early survival of peripheral neurons, including primary afferent nociceptors, but later in development, sensory neurons lose their dependence on NGF for survival but retain expression of its high-affinity receptor-TrkA [30]. It is the small-diameter primary afferent nociceptors (C and A-delta) that continue to express the majority of TrkA receptors [30], and therefore changes in the endogenous or exogenous production of NGF can have profound effects on nociceptor phenotype [32]. End-target tissues of nociceptive terminals, such as epidermal fibroblasts and keratinocytes, are associated with increased NGF production under inflammatory and injury conditions. Together, these elements serve as important signaling pathways for the subsequent perception of pain and hyperalgesia [33]. Therefore, long-term exposure of nociceptive terminals to increased levels of NGF can result in persistent phenotypic changes in the repertoire of nociceptive transducing elements. For example, NGF induces an increase in the mRNA and protein encoding preprotachykinin, the precursor of substance P, and calcitonin gene-related peptide (CGRP), two peptides associated with nociception [34]. Elevated levels of NGF following tissue injury are linked to neuroplastic changes in nociceptor physiology and gene expression [12, 35-39]. Moreover, increased levels of NGF can drive the over-expression of the pain receptors TRPV1 and TRPA1 [12, 13, 31, 40].

NGF is synthesized and secreted by a wide variety of cell types, including peripheral blood mononuclear cells such as macrophages that are known to migrate to sites of tissue injury, including sensory ganglion. Schwann cells are innate immune cells within sensory ganglion and can mimic the function of microglia found in the central nervous system. Both cell types can initiate and regulate local immune responses through the expression of pattern recognition receptors and direct local and adaptive immune responses [5]. Through these exogenous and endogenous linkages, it is proposed that early NGF effects (seconds - minutes) involve direct post-translational modification of TRPV1 in the nerve terminal, leading to a lower threshold of activation [41], whereas long-term effects (days - weeks - months) induce a persistent mechanical hypersensitivity in both mice (and humans) [42] that is mediated in nociceptors through the expression of the high-affinity NGF receptor - TrkA [30, 43].

## 4 SP4 GENE TRANSCRIPTION

TRPV1 is necessary for the development and persistence of inflammatory thermal hyperalgesia and is implicated in other experimental and clinical pain states [44-49]. We hypothesized that the persistence of painful hypersensitivity induced by inflammation and neuroinflammation was dependent on sustained, aberrant gene expression in nociceptors. In pursuit of this idea, we have characterized transcriptional control elements responsible for the expression of TRPV1 in nociceptors [38, 50]. This analysis revealed a dual promoter system that is positively regulated by NGF [38]. The proximal P2 promoter contains a GC-rich DNA binding domain that is required for TRPV1 transcriptional activity. Two members of the Sp1-like transcription factor family, Sp4 and, to a lesser extent, Sp1, bind to the TRPV1 P2 promoter domain, supporting the idea that factor Sp4 positively regulates TRPV1 expression [39].

Sp is a member of the Sp1-like transcription factor family and is predominantly expressed in neurons [51-54]. Sp4 has been linked to various neuronal processes, including signaling [55-58], energy production [59, 60], and conditions such as bipolar disorder [61-63]. Members of the Sp1-like transcription factor family, such as Sp4, are distinguished by their ability to bind GC-box regulatory domains. While Sp1-like members share certain common characteristics of binding to GC-rich targets *in vitro,* they display remarkable diversity for gene-specific regulation *in vivo* [53, 64, 65]. For example, Sp4 has been shown to regulate NMDAR1 (NR1) protein in the brain and cerebellar granule neurons, although its relationship in the spinal cord is thus far unreported [57]. Interestingly, NMDA-mediated cellular depolarization decreases Sp4 phosphorylation, resulting in an increase in Sp4 promoter binding that may represent a model whereby nociceptive input drives spinal (NMDA) activation and in turn, regulates Sp4 activation [55].

While Sp4 was known to be expressed in the central nervous system [66-69], we established the pattern of expression of Sp4 in dorsal root ganglion (DRG), finding Sp4 is expressed in the smaller-sized DRG neurons with nociceptive features [70]. Importantly, we demonstrated in genetically modified mice that a 50% decrease of Sp4 reverses models of persistent inflammatory thermal hyperalgesia and mechanical hypersensitivity following hind-paw injection of NGF or systemic treatment of mice with the chemotherapy agent - oxaliplatin [70]. Sp1-like factors such as Sp4 can undergo post-translational modification based on extracellular signaling (*e.g*., NGF-TrkA). This may explain how inflammation and neuroinflammation-induced transcription factor modifications result in the activation or repression of gene transcription in nociceptors [53, 67, 71, 72]. A, transcriptional model of NGF-induced increase in TRPV1 gene expression in nociceptors is illustrated in Fig. (**2**).

Based on the finding that genetic reduction of Sp4 reversed a model of persistent pain dependent on TRPV1, we investigated the role of Sp4 on the expression of another nociceptive ligand-gated ion channel, TRPA1, shown to mediate inflammatory hypersensitivity [24, 73]. We found that a 50% reduction in Sp4^+/-^ mice decreased TRPA1 mRNA expression in DRG [70]. To determine the functional consequence of a reduction of Sp4 on TRPA1 activity in nociceptors, we measured changes in intracellular calcium in primary cultures of DRG neurons in response to the TRPA1 agonist, allyl isothiocyanate (AITC). As shown in Fig. (**3**), DRG neurons derived from Sp4^+/-^ mice had a reduced magnitude and a number of AITC-induced responses. Taken together, these factors support the hypothesis that Sp4 expressed in nociceptors serves as a master regulator of nociceptive pain transduction and contributes to the persistence of hyperalgesic states.

## 5 SP4 AND ACUTE TO CHRONIC PAIN

We have investigated the hypothesis that Sp4 directs a nociceptive transcriptome in DRG that is primarily responsible for the persistence of hypersensitive states without affecting baseline detection of noxious stimuli. Under this paradigm, Sp4 regulates a network of genes in DRG that are either up- or down-regulated under conditions of inflammation or neurotoxicity and, together, direct painful hypersensitivity. This idea is supported by our study of Sp4^+/-^ mice that revealed a reduction and spontaneous reversal of persistent hypersensitivity, despite ongoing evidence of peripheral inflammation [70]. We surmised that Sp4^+/-^ knockdown mice can be utilized as a tool to study the transcriptomics underlying the genes and pathways responsible for the reversal of persistent hyperalgesic states.

**Fig. (3):**
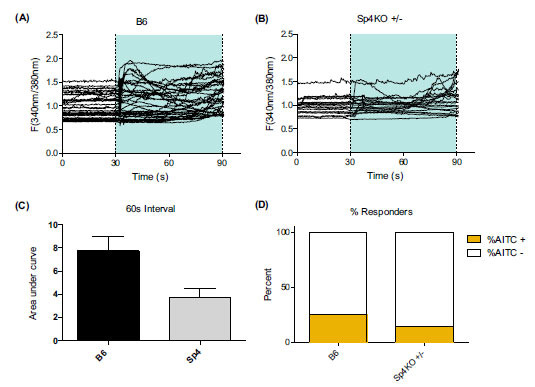
Sp4^+/-^ mice have decreased magnitude and number of DRG neurons responsive to TRPA1 agonist mustard oil: allyl isothiocyanate (AITC). Cultured DRG neurons from Sp4^+/-^ and wild-type C57/Bl6 mice were treated with AITC (20uM), and intracellular calcium responses were recorded over a 60-second period after the addition of the agonist. (**A**, **B**) AITC induced increases in intracellular calcium for control (**A**) and Sp4^+/-^ (**B**) mice. The addition of AITC occurred at 30 seconds, and responses were recorded over a 60-second period (shaded areas). (**C**) DRG neurons from Sp4^+/-^ mice showed a 2-fold reduction in magnitude (area under the curve) of TRPA1 intracellular calcium responses compared to Wt. (**D**) The relative number of neurons showing a response to AITC is reduced in Sp4^+/-^ mouse DRGs. 26% of wild-type neurons showed AITC-induced responses compared to 15% of Sp4^+/-^ neurons. Two wild types and two Sp4^+/-^ animals were used over two independent experiments, generating a total of 141 wild-type cells and 114 Sp4^+/-^ cells counted. Of the total cells, 37 wild-types and 17 Sp4^+/-^ responded. Data are presented as mean area ± SEM of 3 mice per group.

**Fig. (4):**
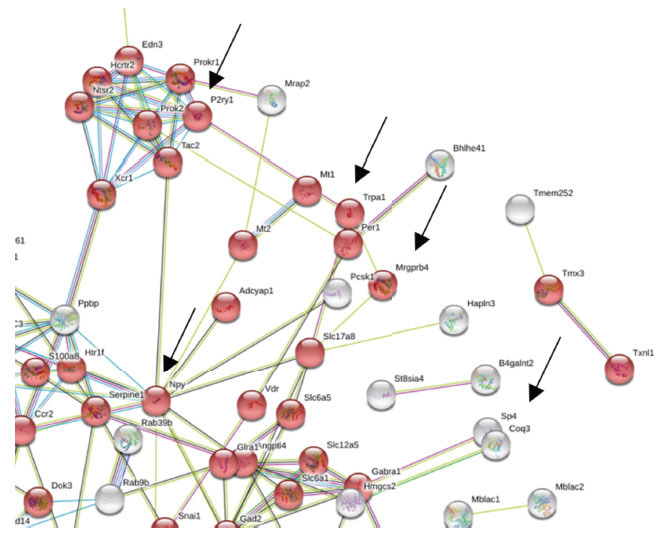
Network map illustrating the connectivity of predicted functional partners for Sp4 differentially expressed genes in DRG. General analysis of gene expression data derived from RNAseq analysis was based on best practices [81, 82]. The significance of candidate Sp4 target genes was set at a *p*-value of 0.05 and transcriptome-wide, at a false discovery rate of 5% under the BH procedure [83]. Nodes represent all proteins produced by a single protein-coding gene locus. Edges represent specific or meaningful associations [84]. Node color: response to stimulus (GO:0050896) genes (red), Muscle system process (GO:003012) genes (blue), and the second shell of interactors (white). The color of the edges connecting the nodes represents the types of evidence supporting the connections: predicted gene neighborhood (green), predicted gene fusions (red), known interactions from experimental evidence (pink), co-expression (black), and text-mining (green). Examples of Sp4-dependent genes with nociceptive function found to be down-regulated: (Blue arrows) P2RY1, TRPA1, Mrgprb4, Sp4; or up-regulated: (Red arrow) Npy. Sp4^+/-^ (n=2) and wild type (n=3) mice. Only a portion of the PPIN is shown.

A transcriptomic study was performed by analyzing differences in lumbar DRG-expressed genes derived from wild-type *versus* Sp4^+/-^ heterozygous knockdown mice [70]. Measurement of differentially expressed genes (DEGs) from total RNA in DRG was performed using RNAseq. The resulting network of 125 up-regulated DEGs was enriched for known or predicted protein-protein interaction suggesting a functional association between them. We identified TRPA1 and Mrgprb4 (Fig. **4**) as components of a Reactome pathway and validated the downregulation of TRPA1 in Sp4^+/-^ DRG by qRT-PCR [70] and functionally in DRG neurons by calcium imaging (Fig. **3**). TRPA1 and Mrgprb4 are associated with mechanosensitivity in DRG [74, 75] and serve experimentally as markers of mechano-transducing sensory neurons. This finding is concordant with a reversal in NGF- and oxaliplatin-induced mechanical hypersensitivity in Sp4^+/-^ mice [70]. In contrast, Npy (neuropeptide y), was found to be increased in Sp4^+/-^ DRG and has been reported to be increased in DRG neurons in response to neuropathic conditions [76]. Taken together, these initial findings support our hypothesis that Sp4 serves a broader regulatory role across a nociceptive transcriptome and plays a critical role in sustaining the persistence of pain arising from inflammation and neuroinflammation.

While our understanding of the mechanisms transducing acute pain in sensory neurons has made tremendous advances, how acute pain becomes persistent or chronic remains elusive and a barrier to the development of successful therapeutic strategies. This review has advanced the hypothesis that members of the Sp1-like transcription factor family, specifically Sp4, are intricately involved in sustaining painful hypersensitivity through its regulation of a nociceptive transcriptome. It remains to be investigated what role Sp4 serves in non-neuronal cells such as Schwann cells, satellite glia, and macrophages [77]. While the expression of pain-transducing ion channels such as TRPV1 and TRPA1 are regulated by Sp4, initial transcriptomic analysis allows us to speculate that a broader network of genes derived from both neuronal and non-neuronal cells contribute to sustaining chronic pain. Under conditions of neuroinflammation, factors such as NGF can direct both post-translation sensitization of nociceptive channels and increase expression of pain-transducing elements in an Sp4-dependent manner. Other emerging mediators and receptor families that contribute to neuroinflammation and hypersensitivity include toll-like receptor signaling [78], chemokines CCL2 and its receptor CCR2 [79] and other signaling molecules from non-neuronal cells [80]. Together these elements appear to form a neuro-immune network underlying neuroinflammation. Through a persistent cycle of nociceptor activation and the production of inflammatory mediators, these components sustain persistent hypersensitivity.

## CONCLUSION

Current therapies for the treatment of chronic pain are insufficient and may be associated with significant adverse events and risks. Opioids, although effective for acute pain at rest, and advancing cancer pain, may poorly control chronic pain and/or hypersensitivity associated with movement [85, 86]. Alternately, the investigation of therapeutics that can modulate nociceptive transcription may hold promise. We have found that the Sp1-like transcriptional inhibitor mithramycin - A decreased TRPV1 promoter activity in transfected PC12 cells [39], and mithramycin dose-dependently decreased TRPV1 mRNA, TRPV1-immunoreactive protein expression and the number of capsaicin-responding DRG neurons in primary culture [87]. Moreover, inhibition of Sp1 by mithramycin was reported by others to block neuropathic pain through down-regulation of the calcium channel alpha2-delta1, a site of action of the anti-neuropathic drug gabapentin [88]. Taken together, these findings support the notion that clinically useful transcription-based inhibitors may one day be developed to effectively reverse or prevent the transition from acute to chronic pain.

## Figures and Tables

**Fig. (1):**
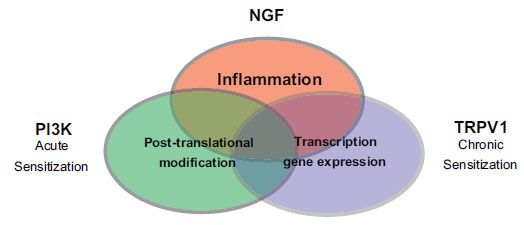
Overlapping mechanisms drive the transition from acute to chronic pain. Peripheral inflammation/neuro-inflammation induced by tissue injury, disease, and medical interventions, triggers the production and release of inflammatory mediators such as nerve growth factor (NFG). Specialized sensory neurons that function to detect painful stimuli (nociceptors) can bind NGF and direct acute changes in pain-transducing ion channels by direct activation and post-translational modifications. The resulting reduction in nociceptive activation thresholds establishes a state of nociceptor sensitization and painful hypersensitivity to chemical, thermal, and mechanical stimuli. Depending on the mechanism and extent of tissuenerve injury, longer-term (Chronic) increases in pain-transducing ion channels, such as TRPV1, are driven by the dysregulation of gene transcription and likely amplify and sustain the persistence of pain.

**Fig. (2):**
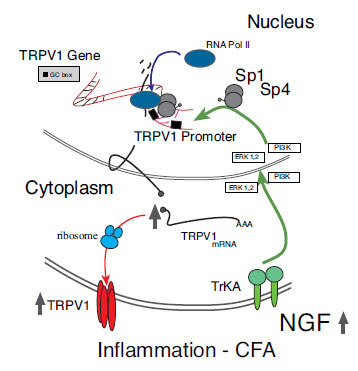
Inflammation-induced NGF directs increased expression of TRPV1 by transcription factor Sp4 Experimentally- induced peripheral inflammation (complete Freund’s adjuvant - CFA) induces local production of NGF. Signaling through high-affinity NGF receptors (TrkA) expressed on the terminals of nociceptive sensory neurons results in activating transcription factors Sp4 and Sp1 and binding to TRPV1 promoter regions. Increased transcription at the TRPV1 promoter site drives the pathophysiologic expression of TRPV1 in sensory neurons.

## LIST OF ABBREVIATIONS

CCL2: C-C Motif Chemokine Ligand
2 CCR2: C-C Motif Chemokine Receptor 2
CGRP: Calcitonin Gene-related Peptide
NT-3: Neurotrophin-3
NT-4: Neurotrophin-4
Sp1: Specificity Protein 1 Transcription Factor
Sp4: Specificity Protein 4 Transcription Factor
RNAseq: RNA Sequencing
TrkA: High-affinity Nerve Growth Factor Receptor
TRPA1: Transient Receptor Potential Cation Channel, Subfamily A, Member 1
TRPV1: Transient Receptor Potential Cation Channel, Subfamily V, Member 1

## References

[1] Institute of Medicine (US) Committee on Advancing Pain Research, Care, and Education (2011). Relieving Pain in America: A Blueprint for Transforming Prevention, Care, Education, and Research.

[2] Kehlet H., Jensen T.S., Woolf C.J. (2006). Persistent postsurgical pain: Risk factors and prevention.. Lancet.

[3] Basbaum A.I., Bautista D.M., Scherrer G., Julius D. (2009). Cellular and molecular mechanisms of pain.. Cell.

[4] Reichling D.B., Green P.G., Levine J.D. (2013). The fundamental unit of pain is the cell.. Pain.

[5] Guan Z., Hellman J., Schumacher M. (2016). Contemporary views on inflammatory pain mechanisms: Trping over innate and microglial pathways.. F1000 Res..

[6] Apkarian A.V., Bushnell M.C., Treede R.D., Zubieta J.K. (2005). Human brain mechanisms of pain perception and regulation in health and disease.. Eur. J. Pain.

[7] Dworkin R.H., Turk D.C., Basch E., Berger A., Cleeland C., Farrar J.T., Haythornthwaite J.A., Jensen M.P., Kerns R.D., Markman J., Porter L., Raja S.N., Ross E., Todd K., Wallace M., Woolf C.J. (2011). Considerations for extrapolating evidence of acute and chronic pain analgesic efficacy.. Pain.

[8] De Felice M., Sanoja R., Wang R., Vera-Portocarrero L., Oyarzo J., King T., Ossipov M.H., Vanderah T.W., Lai J., Dussor G.O., Fields H.L., Price T.J., Porreca F. (2011). Engagement of descending inhibition from the rostral ventromedial medulla protects against chronic neuropathic pain.. Pain.

[9] Piomelli D., Sasso O. (2014). Peripheral gating of pain signals by endogenous lipid mediators.. Nat. Neurosci..

[10] Sexton J.E., Vernon J., Wood J.N. (2014). TRPs and Pain.. Handb. Exp. Pharmacol..

[11] Amaya F., Oh-hashi K., Naruse Y., Iijima N., Ueda M., Shimosato G., Tominaga M., Tanaka Y., Tanaka M. (2003). Local inflammation increases vanilloid receptor 1 expression within distinct subgroups of DRG neurons.. Brain Res..

[12] Amaya F., Shimosato G., Nagano M., Ueda M., Hashimoto S., Tanaka Y., Suzuki H., Tanaka M. (2004). NGF and GDNF differentially regulate TRPV1 expression that contributes to development of inflammatory thermal hyperalgesia.. Eur. J. Neurosci..

[13] Petruska J.C., Mendell L.M. (2004). The many functions of nerve growth factor: Multiple actions on nociceptors.. Neurosci. Lett..

[14] Anand U., Otto W.R., Facer P., Zebda N., Selmer I., Gunthorpe M.J., Chessell I.P., Sinisi M., Birch R., Anand P. (2008). TRPA1 receptor localisation in the human peripheral nervous system and functional studies in cultured human and rat sensory neurons.. Neurosci. Lett..

[15] Andersson D.A., Gentry C., Moss S., Bevan S. (2008). Transient receptor potential A1 is a sensory receptor for multiple products of oxidative stress.. J. Neurosci..

[16] Asgar J., Zhang Y., Saloman J.L., Wang S., Chung M.K., Ro J.Y. (2015). The role of TRPA1 in muscle pain and mechanical hypersensitivity under inflammatory conditions in rats.. Neuroscience.

[17] Bandell M., Story G.M., Hwang S.W., Viswanath V., Eid S.R., Petrus M.J., Earley T.J., Patapoutian A. (2004). Noxious cold ion channel TRPA1 is activated by pungent compounds and bradykinin.. Neuron.

[18] Bautista D.M., Jordt S.E., Nikai T., Tsuruda P.R., Read A.J., Poblete J., Yamoah E.N., Basbaum A.I., Julius D. (2006). TRPA1 mediates the inflammatory actions of environmental irritants and proalgesic agents.. Cell.

[19] Bautista D.M., Pellegrino M., Tsunozaki M. (2013). TRPA1: A gatekeeper for inflammation.. Annu. Rev. Physiol..

[20] Bell J.T., Loomis A.K., Butcher L.M., Gao F., Zhang B., Hyde C.L., Sun J., Wu H., Ward K., Harris J., Scollen S., Davies M.N., Schalkwyk L.C., Mill J., Ahmadi K.R., Ainali C., Barrett A., Bataille V., Bell J.T., Buil A., Deloukas P., Dermitzakis E.T., Dimas A.S., Durbin R., Glass D., Grundberg E., Hassanali N., Hedman A.K., Ingle C., Knowles D., Krestyaninova M., Lindgren C.M., Lowe C.E., McCarthy M.I., Meduri E., di Meglio P., Min J.L., Montgomery S.B., Nestle F.O., Nica A.C., Nisbet J., O’Rahilly S., Parts L., Potter S., Sekowska M., Shin S-Y., Small K.S., Soranzo N., Spector T.D., Surdulescu G., Travers M.E., Tsaprouni L., Tsoka S., Wilk A., Yang T-P., Zondervan K.T., Williams F.M.K., Li N., Deloukas P., Beck S., McMahon S.B., Wang J., John S.L., Spector T.D. (2014). Differential methylation of the TRPA1 promoter in pain sensitivity.. Nat. Commun..

[21] Cattaruzza F., Johnson C., Leggit A., Grady E., Schenk A.K., Cevikbas F., Cedron W., Bondada S., Kirkwood R., Malone B., Steinhoff M., Bunnett N., Kirkwood K.S. (2013). Transient receptor potential ankyrin 1 mediates chronic pancreatitis pain in mice.. Am. J. Physiol. Gastrointest. Liver Physiol..

[22] da Costa D.S.M., Meotti F.C., Andrade E.L., Leal P.C., Motta E.M., Calixto J.B. (2010). The involvement of the transient receptor potential A1 (TRPA1) in the maintenance of mechanical and cold hyperalgesia in persistent inflammation.. Pain.

[23] Diogenes A., Akopian A.N., Hargreaves K.M. (2007). NGF up-regulates TRPA1: Implications for orofacial pain.. J. Dent. Res..

[24] Gregus A.M., Doolen S., Dumlao D.S., Buczynski M.W., Takasusuki T., Fitzsimmons B.L., Hua X.Y., Taylor B.K., Dennis E.A., Yaksh T.L. (2012). Spinal 12-lipoxygenase-derived hepoxilin A3 contributes to inflammatory hyperalgesia via activation of TRPV1 and TRPA1 receptors.. Proc. Natl. Acad. Sci..

[25] Zappia K.J., O’Hara C.L., Moehring F., Kwan K.Y., Stucky C.L. (2017). Sensory neuron-specific deletion of TRPA1 results in mechanical cutaneous sensory deficits.. eNeuro.

[26] Bonnie R.J. (2017). Pain Management and the Opioid Epidemic: Balancing Societal and Individual Benefits and Risks of Prescription Opioid Use.

[27] Woolf C.J. (2011). Central sensitization: Implications for the diagnosis and treatment of pain.. Pain.

[28] McGreevy K., Bottros M.M., Raja S.N. (2011). Preventing chronic pain following acute pain: Risk factors, preventive strategies, and their efficacy.. Eur. J. Pain Suppl..

[29] Lewin G.R., Mendell L.M. (1994). Regulation of cutaneous C-fiber heat nociceptors by nerve growth factor in the developing rat.. J. Neurophysiol..

[30] Andreev N.Y., Dimitrieva N., Koltzenburg M., McMahon S.B. (1995). Peripheral administration of nerve growth factor in the adult rat produces a thermal hyperalgesia that requires the presence of sympathetic post-ganglionic neurones.. Pain.

[31] Koltzenburg M. (1999). The changing sensitivity in the life of the nociceptor.. Pain.

[32] Michael G.J., Priestley J.V. (1999). Differential expression of the mRNA for the vanilloid receptor subtype 1 in cells of the adult rat dorsal root and nodose ganglia and its downregulation by axotomy.. J. Neurosci..

[33] Woolf C.J., Costigan M. (1999). Transcriptional and posttranslational plasticity and the generation of inflammatory pain.. Proc. Natl. Acad. Sci..

[34] Lindsay R.M., Harmar A.J. (1989). Nerve growth factor regulates expression of neuropeptide genes in adult sensory neurons.. Nature.

[35] McMahon S.B. (1996). NGF as a mediator of inflammatory pain.. Philos. Trans. R. Soc. Lond. B Biol. Sci..

[36] Ji R.R., Samad T.A., Jin S.X., Schmoll R., Woolf C.J. (2002). p38 MAPK activation by NGF in primary sensory neurons after inflammation increases TRPV1 levels and maintains heat hyperalgesia.. Neuron.

[37] Zhang X., Huang J., McNaughton P.A. (2005). NGF rapidly increases membrane expression of TRPV1 heat-gated ion channels.. EMBO J..

[38] Xue Q., Jong B., Chen T., Schumacher M.A. (2007). Transcription of rat TRPV1 utilizes a dual promoter system that is positively regulated by nerve growth factor.. J. Neurochem..

[39] Chu C., Zavala K., Fahimi A., Lee J., Xue Q., Eilers H., Schumacher M.A. (2011). Transcription factors Sp1 and Sp4 regulate TRPV1 gene expression in rat sensory neurons.. Mol. Pain.

[40] Bonnington J.K., McNaughton P.A. (2003). Signalling pathways involved in the sensitisation of mouse nociceptive neurones by nerve growth factor.. J. Physiol..

[41] Chuang H., Prescott E.D., Kong H., Shields S., Jordt S.E., Basbaum A.I., Chao M.V., Julius D. (2001). Bradykinin and nerve growth factor release the capsaicin receptor from PtdIns(4,5)P2-mediated inhibition.. Nature.

[42] Rukwied R., Mayer A., Kluschina O., Obreja O., Schley M., Schmelz M. (2010). NGF induces non-inflammatory localized and lasting mechanical and thermal hypersensitivity in human skin.. Pain.

[43] Amann R., Schuligoi R., Herzeg G., Donnerer J. (1996). Intraplantar injection of nerve growth factor into the rat hind paw: Local edema and effects on thermal nociceptive threshold.. Pain.

[44] Caterina M.J., Schumacher M.A., Tominaga M., Rosen T.A., Levine J.D., Julius D. (1997). The capsaicin receptor: A heat-activated ion channel in the pain pathway.. Nature.

[45] Caterina M.J., Leffler A., Malmberg A.B., Martin W.J., Trafton J., Petersen-Zeitz K.R., Koltzenburg M., Basbaum A.I., Julius D. (2000). Impaired nociception and pain sensation in mice lacking the capsaicin receptor.. Science.

[46] Davis J.B., Gray J., Gunthorpe M.J., Hatcher J.P., Davey P.T., Overend P., Harries M.H., Latcham J., Clapham C., Atkinson K., Hughes S.A., Rance K., Grau E., Harper A.J., Pugh P.L., Rogers D.C., Bingham S., Randall A., Sheardown S.A. (2000). Vanilloid receptor-1 is essential for inflammatory thermal hyperalgesia.. Nature.

[47] Schumacher M.A. (2010). Transient receptor potential channels in pain and inflammation: Therapeutic opportunities.. Pain Pract..

[48] Blackshaw L.A. (2014). Transient receptor potential cation channels in visceral sensory pathways.. Br. J. Pharmacol..

[49] Lawton S.K., Xu F., Tran A., Wong E., Prakash A., Schumacher M., Hellman J., Wilhelmsen K. (2017). N -arachidonoyl dopamine modulates acute systemic inflammation via nonhematopoietic TRPV1.. J. Immunol..

[50] Xue Q., Yu Y., Trilk S.L., Jong B.E., Schumacher M.A. (2001). The genomic organization of the gene encoding the vanilloid receptor: Evidence for multiple splice variants.. Genomics.

[51] Supp D.M., Witte D.P., Branford W.W., Smith E.P., Potter S.S. (1996). Sp4, a member of the Sp1-family of zinc finger transcription factors, is required for normal murine growth, viability, and male fertility.. Dev. Biol..

[52] Suske G. (1999). The Sp-family of transcription factors.. Gene.

[53] Bouwman P., Philipsen S. (2002). Regulation of the activity of Sp1-related transcription factors.. Mol. Cell. Endocrinol..

[54] Li L., He S., Sun J.M., Davie J.R. (2004). Gene regulation by Sp1 and Sp3.. Biochem. Cell Biol..

[55] Saia G., Lalonde J., Sun X., Ramos B., Gill G. (2014). Phosphorylation of the transcription factor Sp4 is reduced by NMDA receptor signaling.. J. Neurochem..

[56] Priya A., Johar K., Nair B., Wong-Riley M.T.T. (2014). Specificity protein 4 (Sp4) regulates the transcription of AMPA receptor subunit GluA2 (Gria2).. Biochim. Biophys. Acta Mol. Cell Res..

[57] Sun X., Pinacho R., Saia G., Punko D., Meana J.J., Ramos B., Gill G. (2015). Transcription factor Sp4 regulates expression of nervous wreck 2 to control NMDAR1 levels and dendrite patterning.. Dev. Neurobiol..

[58] Nair B., Johar K., Priya A., Wong-Riley M.T.T. (2016). Specificity protein 4 (Sp4) transcriptionally regulates inhibitory GABAergic receptors in neurons.. Biochim. Biophys. Acta Mol. Cell Res..

[59] Johar K., Priya A., Dhar S., Liu Q., Wong-Riley M.T.T. (2013). Neuron-specific specificity protein 4 bigenomically regulates the transcription of all mitochondria- and nucleus-encoded cytochrome c oxidase subunit genes in neurons.. J. Neurochem..

[60] Johar K., Priya A., Wong-Riley M.T.T. (2014). Regulation of Na +/K + -ATPase by neuron-specific transcription factor Sp4: implication in the tight coupling of energy production, neuronal activity and energy consumption in neurons.. Eur. J. Neurosci..

[61] Zhou X., Tang W., Greenwood T.A., Guo S., He L., Geyer M.A., Kelsoe J.R. (2009). Transcription factor SP4 is a susceptibility gene for bipolar disorder.. PLoS One.

[62] Shi J., Potash J.B., Knowles J.A., Weissman M.M., Coryell W., Scheftner W.A., Lawson W.B., DePaulo J.R., Gejman P.V., Sanders A.R., Johnson J.K., Adams P., Chaudhury S., Jancic D., Evgrafov O., Zvinyatskovskiy A., Ertman N., Gladis M., Neimanas K., Goodell M., Hale N., Ney N., Verma R., Mirel D., Holmans P., Levinson D.F. (2011). Genome-wide association study of recurrent early-onset major depressive disorder.. Mol. Psychiatry.

[63] Pinacho R., Villalmanzo N., Lalonde J., Haro J.M., Meana J.J., Gill G., Ramos B. (2011). The transcription factor SP4 is reduced in postmortem cerebellum of bipolar disorder subjects: control by depolarization and lithium.. Bipolar Disord..

[64] Chang W.C., Chen B.K. (2005). Transcription factor Sp1 functions as an anchor protein in gene transcription of human 12(S)-lipoxygenase.. Biochem. Biophys. Res. Commun..

[65] Zhao C., He X., Tian C., Meng A. (2006). Two GC-rich boxes in huC promoter play distinct roles in controlling its neuronal specific expression in zebrafish embryos.. Biochem. Biophys. Res. Commun..

[66] Zhou X., Qyang Y., Kelsoe J.R., Masliah E., Geyer M.A. (2007). Impaired postnatal development of hippocampal dentate gyrus in Sp4 null mutant mice.. Genes Brain Behav..

[67] Ramos B., Gaudillière B., Bonni A., Gill G. (2007). Transcription factor Sp4 regulates dendritic patterning during cerebellar maturation.. Proc. Natl. Acad. Sci. USA.

[68] Ramos B., Valín A., Sun X., Gill G. (2009). Sp4-dependent repression of neurotrophin-3 limits dendritic branching.. Mol. Cell. Neurosci..

[69] Lerner L.E., Gribanova Y.E., Whitaker L., Knox B.E., Farber D.B. (2002). The rod cGMP-phosphodiesterase beta-subunit promoter is a specific target for Sp4 and is not activated by other Sp proteins or CRX.. J. Biol. Chem..

[70] Sheehan K., Lee J., Chong J., Zavala K., Sharma M., Philipsen S., Maruyama T., Xu Z., Guan Z., Eilers H., Kawamata T., Schumacher M. (2019). Transcription factor Sp4 is required for hyperalgesic state persistence.. PLoS One.

[71] Merchant J.L., Du M., Todisco A. (1999). Sp1 phosphorylation by Erk 2 stimulates DNA binding.. Biochem. Biophys. Res. Commun..

[72] Chu S., Ferro T.J. (2005). Sp1: Regulation of gene expression by phosphorylation.. Gene.

[73] Lennertz R.C., Kossyreva E.A., Smith A.K., Stucky C.L. (2012). TRPA1 mediates mechanical sensitization in nociceptors during inflammation.. PLoS One.

[74] Brierley S.M., Castro J., Harrington A.M., Hughes P.A., Page A.J., Rychkov G.Y., Blackshaw L.A. (2011). TRPA1 contributes to specific mechanically activated currents and sensory neuron mechanical hypersensitivity.. J. Physiol..

[75] Petrus M., Peier A.M., Bandell M., Hwang S.W., Huynh T., Olney N., Jegla T., Patapoutian A. (2007). A role of TRPA1 in mechanical hyperalgesia is revealed by pharmacological inhibition.. Mol. Pain.

[76] (2017). Jerić M.; Vukojević K.; Vuica, A.; Filipović N. Diabetes mellitus influences the expression of NPY and VEGF in neurons of rat trigeminal ganglion.. Neuropeptides.

[77] De Logu F., De Prá S.D.T., de David Antoniazzi C.T., Kudsi S.Q., Ferro P.R., Landini L., Rigo F.K., de Bem Silveira G., Silveira P.C.L., Oliveira S.M., Marini M., Mattei G., Ferreira J., Geppetti P., Nassini R., Trevisan G. (2020). Macrophages and Schwann cell TRPA1 mediate chronic allodynia in a mouse model of complex regional pain syndrome type I.. Brain Behav. Immun..

[78] Liu X.J., Liu T., Chen G., Wang B., Yu X.L., Yin C., Ji R.R. (2016). TLR signaling adaptor protein MyD88 in primary sensory neurons contributes to persistent inflammatory and neuropathic pain and neuroinflammation.. Sci. Rep..

[79] Dansereau M.A., Midavaine É., Bégin-Lavallée V., Belkouch M., Beaudet N., Longpré J.M., Mélik-Parsadaniantz S., Sarret P. (2021). Mechanistic insights into the role of the chemokine CCL2/CCR2 axis in dorsal root ganglia to peripheral inflammation and pain hypersensitivity.. J. Neuroinflammation.

[80] Ji R.R., Chamessian A., Zhang Y.Q. (2016). Pain regulation by non-neuronal cells and inflammation.. Science.

[81] Conesa A., Madrigal P., Tarazona S., Gomez-Cabrero D., Cervera A., McPherson A. (2016). Szcześniak, M.W.; Gaffney, D.J.; Elo, L.L.; Zhang, X.; Mortazavi, A. A survey of best practices for RNA-seq data analysis.. Genome Biol..

[82] Kukurba K.R., Montgomery S.B. (2015). RNA sequencing and analysis.. Cold Spring Harb. Protoc..

[83] Hochberg Y., Benjamini Y. (1990). More powerful procedures for multiple significance testing.. Stat. Med..

[84] Kober K.M., Schumacher M., Conley Y.P., Topp K., Mazor M., Hammer M.J., Paul S.M., Levine J.D., Miaskowski C. (2019). Signaling pathways and gene co-expression modules associated with cytoskeleton and axon morphology in breast cancer survivors with chronic paclitaxel-induced peripheral neuropathy.. Mol. Pain.

[85] Dowell D., Haegerich T.M., Chou R. (2016). CDC guideline for prescribing opioids for chronic pain—United States, 2016.. JAMA.

[86] Els C., Jackson T.D., Hagtvedt R., Kunyk D., Sonnenberg B., Lappi V.G., Straube S. (2017). High-dose opioids for chronic non-cancer pain: An overview of Cochrane Reviews.. Cochrane Libr..

[87] Zavala K., Lee J., Chong J., Sharma M., Eilers H., Schumacher M.A. (2014). The anticancer antibiotic mithramycin-A inhibits TRPV1 expression in dorsal root ganglion neurons.. Neurosci. Lett..

[88] Gómez K., Sandoval A., Barragán-Iglesias P., Granados-Soto V., Delgado-Lezama R., Felix R., González-Ramírez R. (2019). Transcription factor Sp1 regulates the expression of calcium channel α2δ-1 subunit in neuropathic pain.. Neuroscience.

